# Automatic Assessment of Functional Movement Screening Exercises with Deep Learning Architectures

**DOI:** 10.3390/s23010005

**Published:** 2022-12-20

**Authors:** Andreas Spilz, Michael Munz

**Affiliations:** Research Group Biomechatronics, University of Applied Sciences Ulm, 89081 Ulm, Germany; andreas.spilz@thu.de

**Keywords:** automatic exercise evaluation, deep learning, functional movement screening, IMU, movement analysis

## Abstract

(1) Background: The success of physiotherapy depends on the regular and correct unsupervised performance of movement exercises. A system that automatically evaluates these exercises could increase effectiveness and reduce risk of injury in home based therapy. Previous approaches in this area rarely rely on deep learning methods and do not yet fully use their potential. (2) Methods: Using a measurement system consisting of 17 inertial measurement units, a dataset of four Functional Movement Screening exercises is recorded. Exercise execution is evaluated by physiotherapists using the Functional Movement Screening criteria. This dataset is used to train a neural network that assigns the correct Functional Movement Screening score to an exercise repetition. We use an architecture consisting of convolutional, long-short-term memory and dense layers. Based on this framework, we apply various methods to optimize the performance of the network. For the optimization, we perform an extensive hyperparameter optimization. In addition, we are comparing different convolutional neural network structures that have been specifically adapted for use with inertial measurement data. To test the developed approach, it is trained on the data from different Functional Movement Screening exercises and the performance is compared on unknown data from known and unknown subjects. (3) Results: The evaluation shows that the presented approach is able to classify unknown repetitions correctly. However, the trained network is yet unable to achieve consistent performance on the data of previously unknown subjects. Additionally, it can be seen that the performance of the network differs depending on the exercise it is trained for. (4) Conclusions: The present work shows that the presented deep learning approach is capable of performing complex motion analytic tasks based on inertial measurement unit data. The observed performance degradation on the data of unknown subjects is comparable to publications of other research groups that relied on classical machine learning methods. However, the presented approach can rely on transfer learning methods, which allow to retrain the classifier by means of a few repetitions of an unknown subject. Transfer learning methods could also be used to compensate for performance differences between exercises.

## 1. Introduction

For many physiotherapeutic interventions, the regular and correct execution of condition specific movement exercises is an important component for successful treatment [1,2,3,4,5]. During a treatment, the physiotherapist selects suitable exercises for the patient, creates a workout routine and instructs the patient on the movement patterns. While the patient is exercising, the therapist continuously monitors the patient’s execution and corrects it if necessary. However, it is not reasonable to have professionals supervise every training session of the patient. The time required for the physiotherapist and patient would be disproportionately high and the cost of treatment would increase significantly. Therefore, patients perform the prescribed exercise program without supervision at home. However, without the guidance of the therapist, there may be mistakes in the execution of the exercises, which on the one hand slows down the progress of the treatment, and on the other hand can even aggravate the existing injury or add additional ones [6].

To address this issue, there are already several measuring system based approaches to automatically assess a persons performance on various movement exercises based on the provided data. The different approaches vary in the the possible field of application, the type of measurement systems used and the evaluation techniques applied. The objective of this publication is to build on the existing approaches and develop a system for home-based physiotherapeutic training that can be applied to a wide variety of complex movement exercises and is capable of assessing them in an automatic manner.

In physiotherapeutic treatment, a large amount of exercises are used, which involve a different number of body segments, vary greatly in complexity and train different body functions. An appropriate system for home-based training must therefore be applicable to a wide range of exercises. Many existing approaches for exercise evaluation concentrate on fitness exercises such as squats [7,8,9,10], deadlifts [11] or lunges [12]. The quality of the performed movement is often assessed with a catalog of common movement issues, identified by the authors, which are to be detected in a repetition. However, a system for physiotherapeutic purposes should be able to assess complex acknowledged physiotherapeutic criteria and furthermore be able to do this for several exercises, which focus on different parts of the body. The Functional Movement Screening (FMS) [13,14] is a suitable starting point to develop a corresponding system. FMS is widely used and one of the most popular screening systems in the field of sports physiotherapy. It consists of seven exercises, which focus on different areas of the human musculoskeletal system and systematically determine movement restrictions or weaknesses. Each exercise is assigned a score of “3” (perfect execution), “2” (complete execution with compensation movements), “1” (incomplete execution, even with compensation movements) and 0 (pain occurred). This score is assigned based on a well-defined list of movement characteristics. Research [15] has shown that Cook’s system convinces with high interrater and intrarater reliability values. This circumstance is especially interesting for machine learning applications that depend on unambiguous label information.

As measuring system for automatic movement assessment, depth cameras [8,16,17,18,19] or RGB cameras are often used in combination with human pose estimation algorithms [20,21]. In order to be universally applicable, i.e., to be able to track a person in a wide variety of postures, these systems require several cameras at different positions. This requires a large enough unobstructed area at the user’s home to use these systems, as well as complex calibration routines to provide viable measurement data. These demands on available space, prior knowledge, and resources cannot be expected of a user. A suitable alternative to these camera-based systems are inertial measurement units (IMUs). These units record acceleration, angular velocity and magnetic field strength on three axes. Attached to the body segments, these measuring units can be used to record the kinematic movement of a human being. In addition, they are comparatively inexpensive, do not require additional space and do not cause additional effort for the user during operation in an appropriately designed measurement system. Many other research groups rely on this technology and use setups that vary in the number and position of IMUs, depending on the focus of the movement exercises being considered [7,9,11,22,23]. Approaches with fewer IMUs are easier for a user to utilize, however these are tailored to a specific type of exercise, e.g., knee rehabilitation exercises [23]. The present research question demands a full body IMU tracking setup, as this enables a wide field of application.

For automatic exercise evaluation, classical machine learning methods have been used almost exclusively so far [24]. Current studies mainly rely on methods such as decision tree [7,12,22] and support vector machines [7,23] or ensemble learning methods such as random forest [9,11,18] and AdaBoost [25] to assess different exercises. For example, Wu et al. [25] develop a system which automatically scores FMS exercises using an AdaBoost approach. To accomplish this, 31 hand-crafted features from 11 IMUs are used. With this approach, accuracies of 0.66–0.91 are achieved, depending on the considered FMS exercise.

The major drawback of classical machine learning techniques is that the algorithms rely on hand crafted features. These hand crafted features are manually chosen to fit a specific task based on expert knowledge, experience and general guidelines. Identifying and creating these features is a very time-consuming process, with no guarantee that a suitable set will be created. In contrast, deep learning methods perform automatic feature engineering as part of the learning process, with specialized network structures such as convolutional neural networks (CNNs) or recurrent neural networks (RNNs). Here, the computer does the heavy lifting and learns relevant features on its own, using a general learning procedure [26,27]. Transfer learning is another advantage of deep learning techniques. This technique makes it possible to adapt a model trained on a source task to a target task. This produces good results, although the dataset for the target dataset can be comparatively small. Recent research shows that this technique yields promising results in the analysis of multivariate time series [28]. However, deep learning methods also come with disadvantages. They require significantly more data to deliver meaningful results, the training takes longer and the learned classification boundaries are harder to explain. However, for a generalized, scalable approach to automated assessment of physiotherapy exercises, deep learning is preferable due to the automatic feature engineering and the opportunity to use transfer learning techniques.

In the field of Human Activity Recognition (HAR), there exist classical machine learning techniques as well as deep learning techniques. The former ones use hand-crafted features and classical training algorithms (e.g., [29]). Deep learning techniques, which allow for end-to-end learning setups, are increasingly used in combination with IMU data [30] of human movement. For example, Ordonez et al. [31] have shown that using a combined structure of CNNs and long short term memory (LSTM) layers it is possible to distinguish between different activities using IMU data. This research suggests that neural networks are capable of discriminating human motion using IMU data. That said, HAR and exercise evaluation have some common ground, but they also have significant differences. In HAR, any reasonably long section of an activity represents it. For example, if the data contain only a quarter step cycle, the activity performed is still walking. Accordingly, a general representation of the activity must be learned, so that it can be identified. In contrast, in exercise evaluation, relevant movement elements of an exercise must be recognized, evaluated and these evaluations must be offset against each other to assess the overall movement. To be able to combine the evaluation properly, an entire repetition of an exercise must be considered. This difficulty becomes apparent with the help of an example: A subject performs a squat. Possibly, the subject crouched with perfect execution but made a slight mistake when returning to the standing position and ended the movement with a dangerous incorrect foot posture. In order to evaluate the repetition as a whole, these aspects must be recognized, evaluated and combined properly. Due to the comparable database but the notable differences between the assigned tasks, it is necessary to consider the usability of the deep learning approaches from HAR for the exercise evaluation as well.

However, deep learning techniques are still rarely used for the automatic evaluation of motion tasks based on IMU data. In a first approach, Lee et al. [10] have compared the performance of a random forest approach with an approach based on a combination of a CNN and an LSTM. When classifying a squat into different performance variants based on the data of five IMUs, the deep learning approach achieved significantly better results than the classical approach.

There are several open issues in the use of deep learning methods for exercise evaluation with IMU data that have not yet been investigated:

- variable sequence length: In HAR, it is common to divide a measurement sequence into several windows of equal dimensions [30,31]. This approach is reasonable because most neural networks rely on uniform input dimensions and a segment of a given activity might also contain all the information needed to classify it. To be able to evaluate an exercise repetition, it is not enough to consider only a part of it. The classification system must be able to consider the entire repetition. The duration of multiple repetitions varies of course significantly and accordingly a method has to be developed to create a uniform input representation, without disrupting the temporal context of the data.
- hyperparameter optimization: Hart et al. [24] already noted in their review of various exercise evaluation publications that hyperparameter optimization is often not performed, or not documented, even though the benefits are widely recognized. In particular, for deep learning approaches applied to exercise evaluation, such a process has not yet been documented.
- usage of alternative CNN structures, designed for IMU data: CNNs are originally designed for the analysis of information in image form. By arranging the individual IMU channels as rows of a two-dimensional matrix, CNNs can also analyze this time-varying information. Nevertheless, the two types of information differ significantly. In the case of an image, CNNs perform spatial convolution, whereas in the case of IMU data, convolution occurs over time, the individual measured quantities (acceleration, angular velocity, etc.), and the spatial relationship between IMU units. Recent studies [32,33,34,35] already investigate the influence of different CNN structures on classification performance in HAR. A corresponding investigation for the exercise evaluation has not yet been conducted.
- Existing approaches based on classical machine learning methods often found that the performance of the classifiers used deteriorated significantly when a leave-one-subject-out split (LOSO) was performed [24]. Here, the performance of the classifier is evaluated on the data of a subject that was not included in the training dataset. Such an investigation has not yet taken place for a deep learning approach and should be investigated especially with regard to its use in practice.

In summary, this publication will use the example of FMS exercises to investigate the extent to which deep learning methods can be used to automatically assess different physiotherapeutic exercises. The main contributions of this paper are as follows.

- Introduction of a novel IMU-based dataset for the automatic evaluation of FMS exercises
- Development of a neural network based approach for the automatic evaluation of FMS exercises
- Evaluation of the influence of different hyperparameters and network structures on classification performance
- Performance evaluation of the developed system on different exercises from the FMS

## 2. Materials and Methods

### 2.1. Dataset

To investigate the issue at hand, a suitable dataset was acquired. Within the scope of a measurement campaign, we recorded, processed, and labeled a FMS dataset with 20 subjects of four different exercises. For the successful training of a neural network a dataset with a wide range of variations is required. Additionally, Hart et al. [24] emphasize in their review of various exercise evaluation approaches, that the erroneous examples of movement patterns in a dataset should not be staged. Acted movements can only represent reality to a limited extent and do not provide a sufficient foundation for the learning process. In order to record as diverse unstaged movement patterns as possible, both men and women from different age groups were recruited. During the measurement, subjects must complete an extensive training program designed to provoke fatigue, which in turn leads to deviations from the patient’s standard movement patterns.

The exact specification of the recorded dataset and our methodology is explained in the following.

#### 2.1.1. Exercises

**Figure 1:**
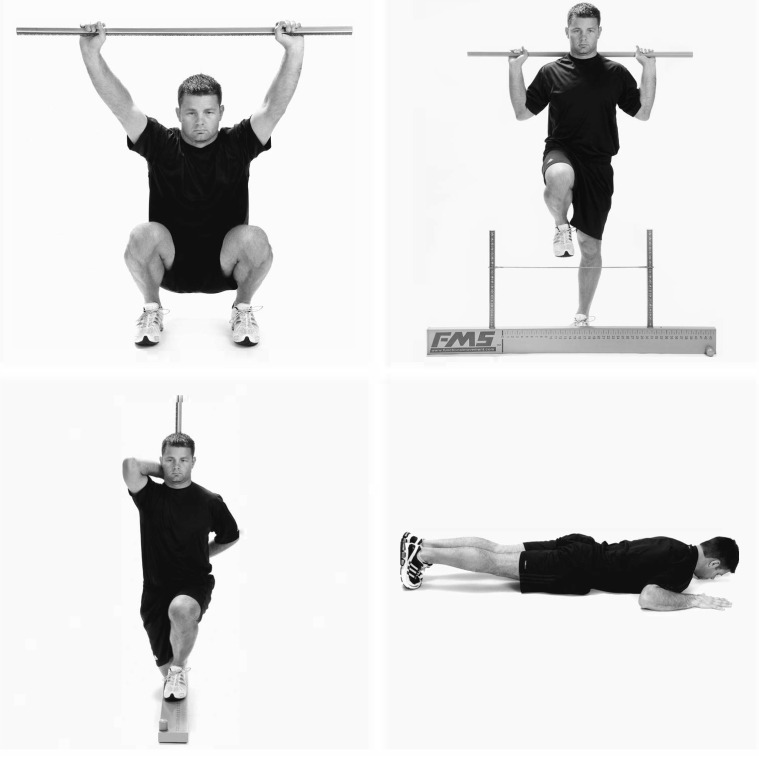
FMS exercises included in the recorded dataset. From left to right, from top to bottom: Deep Squat (DS), Hurdle Step (HS), Inline Lunge (IL) and Trunk Stability Pushup (TSP) [36].

The described dataset contains four exercises from the FMS. Specifically, these are the Deep Squat (DS), Hurdle Step (HS), Inline Lunge (IL), and Trunk Stability Pushup (TSP) exercises. These are modified versions of the commonly known sports exercises squat, pushup and lunge. The HS movement is rather unknown. Here, a subject stands upright in front of a hurdle, the height of which depends on the person’s anatomy. Now the subject must lift one leg over this hurdle and touch the ground on the other side with his heel. A sample image of each exercise can be found in Figure 1. Additional information on the exact movement sequence of each exercise can be found in Cook et al. [36].

#### 2.1.2. Measurement Campaign Population

The exercises presented are performed by a total of 17 healthy volunteers. During recruitment, care is taken to ensure that approximately equal numbers of men (9) and women (11) participate in the study. The age of the volunteers ranges from 24 to 62 years, trying to recruit as balanced as possible from the different age groups (41 ± 13.53 years). The height of the subjects ranges from 160 to 185 cm (173.5 ± 8.3 cm) and the weight ranges from 40 to 110 kg (71.35 ± 14.9 kg). Before the trial begins, each participant signs an informed consent form. The measurement campaign protocol was evaluated and accepted by the ethics committee of the University of Applied Sciences, Ulm, Germany. The measurement campaign presented is registered in the German Clinical Trials Register and can be found under the ID DRKS00027259.

#### 2.1.3. Measurement Setup

Each subject is instrumented with a total of 17 IMUs. We have divided the human body into 17 segments, to each of which an IMU is attached. The number and position of the individual IMUs is based on the *Xsens MVN fullbody* tracking system [37]. The setup definition of *Xsens* seems suitable to us, because *Xsens* has years of experience in professional motion analysis with IMUs and the products are already used in a wide variety of sports. In addition, the systems are already used by other research groups in many areas of motion analysis.

**Figure 2:**
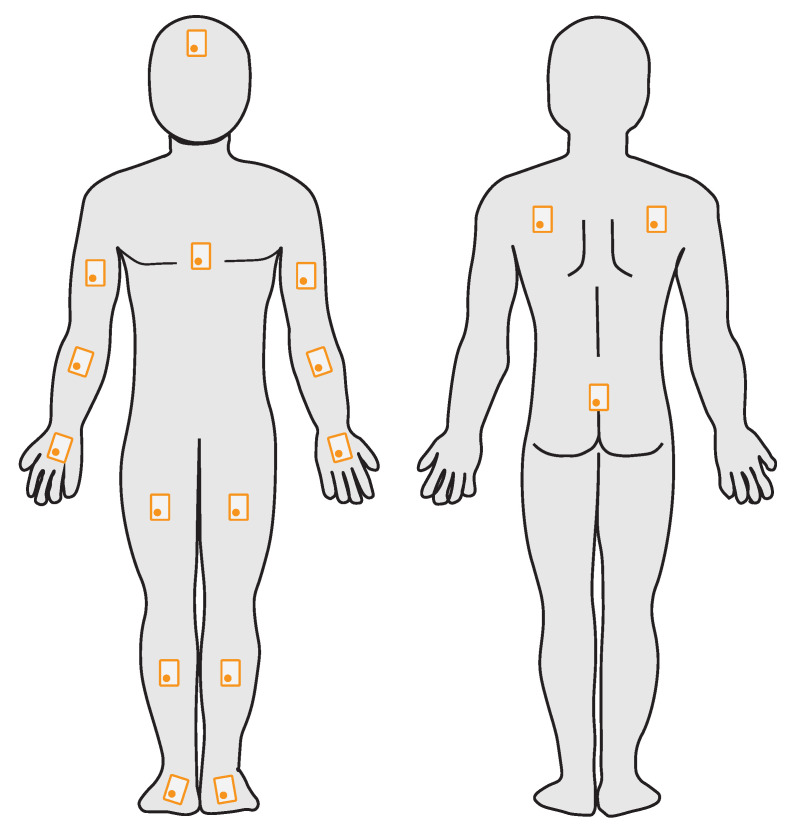
Positioning of the IMUs (orange outline) on the subject.

The exact positioning of the IMUs is shown in Figure 2. The individual IMUs are attached to the subject with elastic straps. The position on a segment is chosen in a manner that minimizes displacement of the IMUs while in motion, e.g., by placing them between muscle bellies. In the described setup we use *Shimmer3* IMUs by *ShimmerSensing*. These IMUs feature a three-axial accelerometer, gyrometer and magnetometer and an air pressure sensor. The individual metrics are recorded with a sampling frequency of 120 Hz on a measuring range of ±16 g (accelerometer), ± $200^{\circ }$ /s (gyrometer), ±49.5 Ga (magnetometer) and 300–1100 hPa (air pressure sensor). To allow for later evaluation of the FMS exercises, the subject is filmed from five perspectives (left, right, top, front and back) with RGB cameras. The *Basler acA640-120gc* is used for this purpose, with a resolution of 640 × 480 px and a sampling rate of 120 Hz.

#### 2.1.4. Procedure

After their arrival, the subjects are informed about the procedure. Subsequently, the IMUs are attached to the previously defined locations. After all measurement systems have been started, a synchronization procedure is performed (for details see Section 2.1.6) and the actual recording starts. The subjects now have to complete a total of three rounds of an exercise circuit. Each round consists of 15 repetitions of DS, 15 repetitions of HS with the right foot, 15 repetitions of HS with the left foot, 15 repetitions of TSP, 15 repetitions of IL with the right foot in front and 15 repetitions with the left foot in front. The 15 repetitions are again divided into five units of 3 repetitions each. After each completed unit, there is a few seconds rest and a longer break after each completed round. Since the overall program is very strenuous, the subjects had the option to stop the experiment at any point. After the experiment is completed, another synchronization procedure is performed.

#### 2.1.5. Labeling

The individual repetitions are evaluated by three physiotherapists on the basis of the video recordings, according to the FMS criteria. However, only the ratings “1” (incomplete execution, even with compensation movements), “2” (complete execution with compensation movements) and “3” (perfect execution) are assigned, since the rating “0” is assigned when pain occurs. This adjustment is made because pain is difficult to detect without input from the subject, and also because the study protocol placed great emphasis on stopping immediately if pain occurred.

Several measures have been implemented to guarantee reliable labels:

- the three physiotherapists are trained uniformly and have equal experience with FMS
- in a test phase, 100 repetitions are labeled by each physiotherapist, subsequently inconsistencies are discussed and corrected
- each repetition is rated by three physiotherapists and the ratings are given independently of each other
- habit effects are avoided by randomly selecting the sample to be rated from all repetitions

To optimize the labeling process, a web application is developed that presents the different video perspectives in an organized way. The application allows the rater to directly enter his feedback for the present repetition and proceed to the next repetition.

After labeling was completed, interrater reliability was assessed. For this purpose, Krippendorff’s alpha [38] is used as an assessment metric. This metric is suitable in the present case because, unlike Cohen’s Kappa for example, it can be used for more than two raters. Moreover, it is suitable for ordinal data, such as the ratings of the FMS.

#### 2.1.6. Postprocessing

After the measurement, the collected data from the cameras and the IMUs is time-synchronized. The cameras were already synchronized during the measurement, with a common trigger signal from a synchronization box. The IMUs are synchronized with each other and with the cameras after the measurement. Since the high number of IMUs is very difficult to synchronize by normal means, an alternative method was developed. This method is based on a precisely defined pulsing magnetic field to which the IMUs are exposed before and after the measurement. With the method described and evaluated in Spilz et al. [39] the individual IMUs can thereby be synchronized with each other with an accuracy of below 2.6 ms. In addition, the magnetic field is controlled by the same box that is responsible for the synchronization of the cameras. Via this box, the two synchronization methods can be put into a temporal context that allows the cameras and the IMUs to be synchronized.

**Figure 3:**
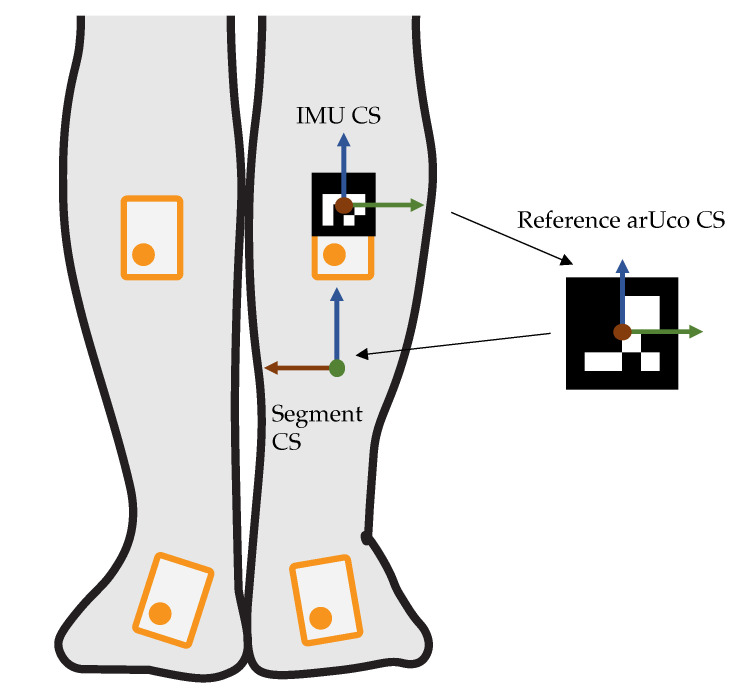
Derivation of the transformation between the IMU CS and the body segment CS. The CS consist of x- (**red**), y- (**green**) and z-axis (**blue**).

Measures are taken to ensure that the IMUs are both attached to the correct body segment and have a consistent orientation to the segment. For this purpose, a so-called arUco marker [40] is attached to each IMU. These markers have a unique ID that can be retrieved from a 2D RGB image. Furthermore, the orientation of the markers relative to the camera can be determined. We attach the IMUs to the subject and then photograph him or her from several perspectives. These images can be used to verify that the individual IMUs are located on the correct segment and also to determine the orientation of the IMUs relative to the body segment. The orientation determination is performed in the following steps: The orientation of the IMU coordinate system (CS) relative to the camera CS is determined via the arUco on the IMUs. In the background of the image, reference arUco markers are placed on the floor and on the wall. These can be used to determine the orientation of the room CS to the camera CS. The subject is clearly positioned in a well-defined posture relative to this room CS. Thus, the orientation of the individual segments to the room CS is known and the orientation of the IMU CS to the body segment CS can be determined (see Figure 3. The resulting transformation is applied to the three-dimensional IMU data, so the measured quantities are in the same segment CS for each subject.

In addition, the positioning of the IMUs was visually monitored during the measurement. In case of a significant displacement, the measurement was excluded.

### 2.2. Architecture and Training

In the presented work, we want to introduce a method to assign a FMS rating of “1”, “2” or “3” to an exercise repetition. This classification task will be performed by a neural network. The structure and the hyperparameters of this network are presented in the following. Our approach is based on a neural network that consists of CNN layers, LSTM layers and dense layers. The input is first passed to the CNN layers, whose task it is to learn relevant features. These features are passed on to the LSTM layers, which analyze temporal dependencies. Finally, the dense layers follow, which are responsible for the actual classification. In the following, we would like to develop the exact structure.

#### 2.2.1. Variable Sequence Length

Due to the differences between HAR and exercise evaluation, there are several factors that must be considered when constructing the input data for the neural network.

First, in contrast to HAR, it is necessary to pass an entire repetition to the network. In HAR, the common approach is to divide a measurement into windows of equal size. Each of these windows is then used as a sample for the dataset. This procedure cannot be adopted for exercise evaluation. The reason lies in the nature of exercise evaluation: In HAR, an unambiguous label can still be assigned to a section, respectively a window, of a performed activity. Even if the window contains e.g., only a fraction of a step, the performed activity and therefore the assigned label, is still “walking”. This approach is not applicable to exercise evaluation, since the existing labels, such as the FMS evaluations, always refer to the entire repetition. If a repetition is labeled as “2” (i.e., errors occurred during the execution), it is not clear which windows of the exercise is responsible for this label. One window might contains the perfect partial execution of a squat, while another window contains the incorrect partial execution responsible for the assigned label “2”. Since the labels only indicate whether errors occur during the execution and not when, the individual windows cannot be provided with unique labels.

Second, the duration of the individual repetitions varies significantly. Typically, problems like this are addressed by resampling the repetitions to a uniform number of time steps [10,24]. However, if this approach is followed, the temporal context in the data is lost. This context is elementary when IMU data are used, e.g., the velocity of the movement depends on the measured acceleration relative to measured time. Therefore, we want to preserve the temporal context and pass it to the neural network.

Concluding, we preprocess the data with the following steps: First, a 2D representation of the data is generated, following the approach of Ordonez et al. [31]. For this purpose the accelerometer and gyrometer channels of the individual IMUs are arranged as rows of a 2D matrix. The channels are sorted IMU by IMU, so that the x, y and z axes of the accelerometer come first, then those of the gyrometer and then the accelerometer channels of the next IMU.

Second, all repetitions are zero-padded to the same length.

**Figure 4:**
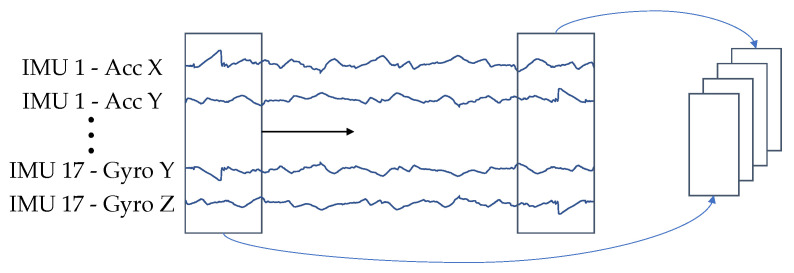
Input representation for the proposed neural network. The individual sensor channels are combined line by line so that they result in an image-like representation.

Third, each repetition is divided into X equal-sized windows without overlap. A graphical illustration of the process can be found in Figure 4.

#### 2.2.2. Basic Architecture

The constructed 3D representation (IMU channels x time steps x windows) is used as input to the neural network. The CNN layers are located within the *TimeDistributed* wrapper of *Tensorflow* 2.5 [41]. This wrapper allows that several temporal slices (windows) of an input are processed by the same layer. As a result the 3D representation can be passed to the network, and each window is processed individually by the same CNN layer. This results in filter weights being learned based on all windows.

Following these CNN layers, the windows are then transferred as steps to the LSTM layer. The LSTM is preceded by a masking layer, which ensures that the windows that contain only 0’s are skipped. Since the LSTM layer can handle any number of time steps, it is possible to process a repetition of any length. With this, the general architecture of the network is defined, now the hyperparameters of the architecture are optimized. The exact network specification is described in the following Section.

#### 2.2.3. Hyperparameter Optimization

**Figure 5:**
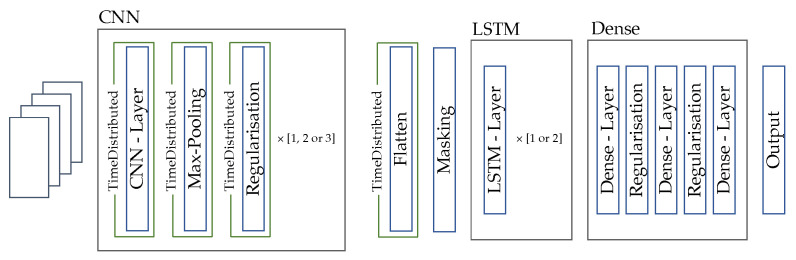
Network architecture of the CNN-LSTM.

To define the exact parameters of the network structure, we perform a hyperparameter optimization. The basic network structure for this optimization is shown in Figure 5.

Table 1 lists the parameters that were varied, along with the range in which they were varied. The parameters were chosen based on our own experience for the presented network structure and supplemented according to the recommendations of Yang et al. [42]. Enclosed are explanations of the terms used:

- activation function: the activation function for each layer except the LSTM layers and the output layer
- CNN-block: refers to a block consisting of a CNN layer, followed by a max-pooling layer and either a dropout or a batch normalization layer
- combination scheme for CNN-blocks: the individual CNN-blocks have different hyperparameters depending on the chosen scheme
- “increasing filters, fixed kernel size”: means the number of filters increases per CNN-block [16, 32, 64] but the kernel size remains the same [(5 × 5), (5 × 5), (5 × 5)]
- “increasing filters, decreasing kernel size”: means the number of filters increases per CNN-block [16, 32, 64] and the kernel size is decreased [(9 × 9), (5 × 5), (3 × 3)]

The remaining hyperparameters are defined as follows. The number of units in every LSTM layer is 256. The number of neurons in the first dense layer is 512, 128 in the second and 3 in the last (output) layer. In the output layer a softmax function is used as activation function, in the LSTM layers we use tanh as activation function and the sigmoid function for the recurrent activation, in all other layers the function that performs best in the optimization is used. The max-pooling operation is performed with a (2 × 2) kernel. Every trainable weight is initialized using the uniform initialization by Glorot. As evaluation metric we use the macro F1-score [43], which is defined as the arithmetic mean of the F1-scores of the individual classes. As error function we use categorical cross-entropy. Adam with a learning rate of 0.0001 is used as optimizer. The datasets are stratified according to the label distribution of the entire dataset. The described network is implemented in *Tensorflow* 2.5.

This optimization is executed using the DS repetitions of the presented dataset. In order to obtain an objective impression of the performance, each parameter combination is tested in a 5-fold cross validation (CV). The dataset is divided into training (74%), validation (16%) and test (20%) set using a stratified shuffle split for each fold.

The channels of the IMUs are arranged and windowed according to Figure 4 to generate a 2D representation. The data was standardized, but separately for accelerometer and gyrometer data.

A total of 90 different parameter combinations is evaluated. For each of these runs, a value for each parameter in Table 1 is randomly chosen. The selection is performed with a *WandB sweeps agent* [44] operating in random mode.

#### 2.2.4. Alternative CNN Structures

**Figure 6:**
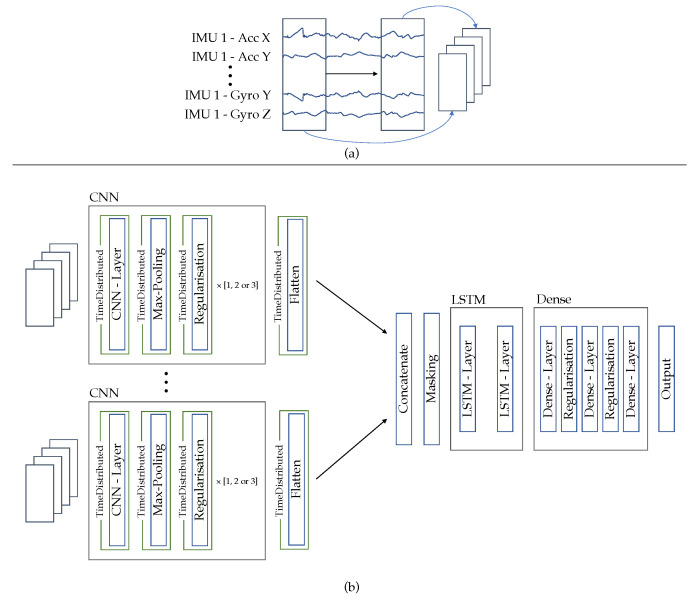
(**a**) Input definition for the tested “IMU-centric” approach. (**b**) Network architecture of the “IMU-centric” approach.

The previously used approach is based on arranging IMU data in two dimensions and applying a two-dimensional convolution to them. There also exist alternative CNN approaches that are specifically designed for IMU data. We want to adapt two existing approaches to our network structure and compare the performance with the previously described “baseline” approach. So three different structures are tested:

- “baseline” approach: The approach described in Section 2.2.1
- “IMU-centric” approach: Following Grzeszick et al. [33] the data of all IMUs are no longer combined to a single 2D representation and processed, but the data of each IMU are processed in a separate network branch. With this approach, the data from the individual IMUs is merged at a later stage and individual filters are learned for each IMU. We are adapting our previous procedure to follow the same approach. For this, the data is zero-padded and windowed as before, but now separated by IMUs (see Figure 6a). The network structure is modified, so that the input data from each IMU are processed by its own network branch, which consists of the CNN-blocks already described. Within these blocks, a channel’s data are convoluted over the time axis, using kernels with the size (1 × 5). Afterwards, the network outputs are concatenated and passed to a masking layer followed by LSTM layers. From here on, the network structure is identical to the previously described architecture (see Figure 6b)
- “channel-centric” approach: Following Münzner et al, the IMU data are convoluted along the time axis channel by channel, but all with the same kernel [32]. This approach ensures that the channels are convoluted along the time axis, with the learned filters being the same for each channel. To adapt this approach, little is changed in the existing framework from Section 2.2.1. Only the kernel size is adapted (1 × 5), the preprocessing of the data and the network structure remain the same

The modified CNN structures may lead to a different feature learning process. This process may now require additional or fewer CNN layers to learn meaningful features. So the different approaches can reach their full potential, we will perform an appropriate series of experiments. Each of the three approaches will be tested with one, two or three CNN-blocks. As in Section 2.2.1, these blocks consist of a CNN layer, a max-pooling layer and a dropout layer. As the number of CNN-blocks increases, the kernel size in each block remains the same, while the number of filters doubles (16/32/64). The kernel of the max-pooling layer always remains the same (2 × 2), as well as the rate of the dropout layer (rate = 0.2). In total, 9 different configurations are tested. A 5-fold cross-validation is performed with each configuration, with a training (74 %) validation (16%) and test (20%) split, stratified by labels. This optimization is executed using the DS repetitions of the presented dataset.

The remaining hyperparameters are chosen on the basis of the results of Section 2.2.3. Accordingly, both LSTM layers have 256 units, and the dense layers have 512, 128 and 3 neurons. In the output layer a softmax function is used as activation function, in the LSTM layers we use tanh as activation function and the sigmoid function for the recurrent activation, in all other layers we use ELU. Every trainable weight was initialized using the uniform initialization by Glorot. As evaluation metric we use the macro F1-score. As error function we use categorical cross-entropy. Adam with a learning rate of 0.0001 is used as optimizer and the batch size is 32.

#### 2.2.5. Evaluation of the Performance on Different Exercises

To this point, all analyses have been performed with the DS repetitions. In the next step, we want to examine whether the developed approach can also be applied to the three other exercises from the presented dataset. For this we use the approach that has performed best so far, i.e., the baseline approach from Section 2.2.4, with three CNN-blocks. The remaining hyperparameters are also adopted from this Section. To obtain an objective overview of the performance, the network is trained with a 5-fold cross validation, as specified in Section 2.2.3. Since the HS and IL exercises can each be performed with an active left or right foot, different variants are considered here: First, for each of the two exercises, one network is trained with the repetitions of the left side only and one network is trained with the repetitions of the right side only. In addition, a network with all repetitions of the left and right side is trained to investigate how the performance of the network is affected.

#### 2.2.6. Evaluation of the Performance on Unknown Subjects

Finally, we would like to investigate how the developed approach performs if repetitions of unknown subjects are classified. For this investigation, we utilize the same network configuration as in Section 2.2.5. However, this time a Leave-One-Subject-Out (LOSO) split is performed, so the test set consists of the data of one subject, which is not part of the training and validation set. The remaining data are divided into training (80% of remaining data) and validation (20% of remaining data) set. This process is performed 10 times, each time with a different subject and is called Leave-One-Subject-Out-Cross-Validation (LOSOCV).

This analysis is performed individually for each of the four exercises in the dataset, whereby HS and IL are again split in three variants: left, right, and combined. In contrast to previous analyses, we use the weighted F1-score [43] instead of the macro F1-score. The reason for this is the label distribution within the data of a single subject. Many subjects received the same score for every performed repetition of a particular exercise. Since these examples represent the entire test dataset in the performed LOSOCV, the macro F1-score would distort the performance evaluation.

## 3. Results

### 3.1. Dataset

**Figure 7:**
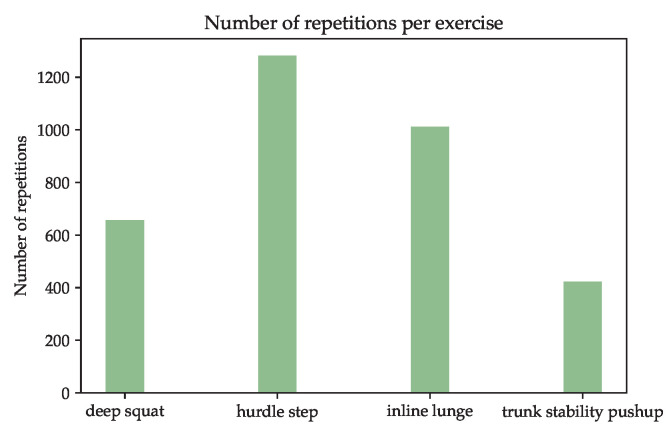
Number ob repetitions per FMS exercise in the presented dataset.

The described study resulted in a dataset with 3374 usable repetitions. The exact number of repetitions per exercise is shown in Figure 7. Not all subjects were physically able to perform the intended number of repetitions, so the experiment was terminated when the subject was no longer able to continue without injury. In particular, the TSP could only be performed correctly by a few subjects due to the physically demanding exercise format. The HS and IL exercises are present more frequently than the other two. The reason for this is, that these exercises should be performed 90 times (45 left, 45 right) per subject, while the other exercises should be performed just 45 times.

To ensure that the labels assigned are reliable, each recurrence is labeled by three physical therapists who independently submitted their ratings. Krippendorff’s alpha for the ratings given is 0.815 for the entire dataset, indicating a high level of agreement among the individual raters [45].

**Figure 8:**
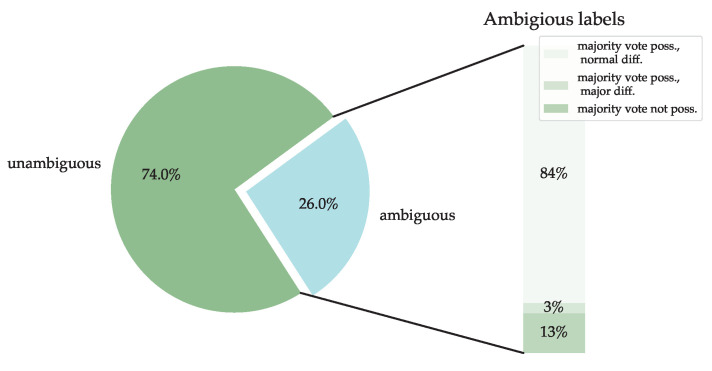
Listing of the different degrees of agreement in the evaluation and their share in the total number of repetitions. For “unambiguous” repetitions, all raters agree. For “ambiguous” repetitions, at least one does not agree with the rest. The category “ambiguous” can be further divided into repetitions where no majority vote is possible, repetitions where a majority vote is possible, but there are major differences in the given ratings and repetitions where a majority vote is possible, but just minor differences in the given ratings.

The final evaluation of a repetition is determined by a majority vote. Figure 8 visualizes the proportions of repetitions that have a certain degree of agreement. The largest proportion of repetitions (74%) is unambiguous, i.e., all raters agree. The remaining 26% of the repetitions show inconsistencies in the rating. This proportion can be further subdivided into repetitions where a majority vote is possible and the discrepant score differs by only one point, and repetitions where a majority vote is possible but the discrepant score differs by more than one point. Additionally, there is a category for repetitions where a majority decision is no longer possible, because all given scores differ from each other. These repetitions are excluded from the dataset for the time being. In total, 3374 repetitions are usable for the development of the algorithms.

**Figure 9:**
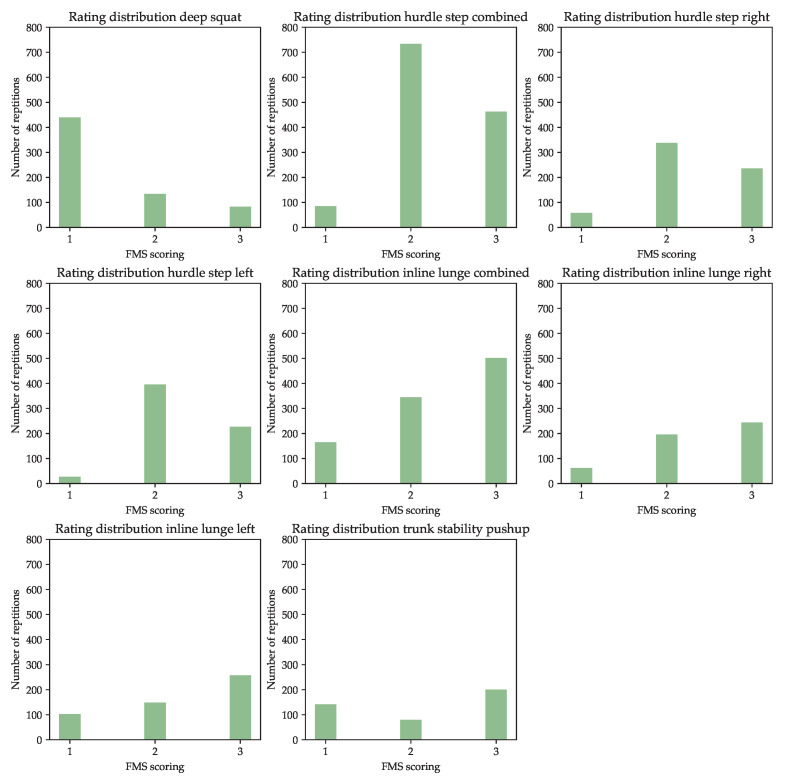
Distribution of the assigned FMS ratings (“1”, “2” or “3”) divided by exercise. HS and IL can be performed with an active left or right foot and are therefore split into, e.g., “HS right” and “HS left”. The distribution over both left and right repetitions is presented with the label “combined”, e.g., HS combined.

The label distribution for each exercise is shown in Figure 9. As can be seen, the label distribution for each exercise is skewed. Interestingly, a different rating is over represented for each exercise. The clearest differences are found in the HS left, where the rating “2” is about 14 times more frequent than the rating 1. The most balanced distribution is found for the TSP, here the ratings “1” and “3” occur only 1.5 times and 2 times as frequently as the rating “2”.

### 3.2. Hyperparameter Optimization

The optimal parameter combination is selected using the averaged performance from the 5-fold CV. Considered is the averaged macro F1-score achieved on the validation dataset. The best performance was achieved by a network with the following hyperparameters: batch size 32, three CNN-blocks with the “increasing filter, fixed kernel size” scheme, dropout with a rate of 0.2 as a regularization technique, and two LSTM layers. This combination results in a macro F1-score of 0.956 on the training set, 0.955 on the validation set, and 0.906 on the test set. In all tested configurations, macro F1-scores were achieved in the ranges of 0.932–0.969 (training set), 0.939–0.955 (validation set), and 0.867–0.906 (test set).

### 3.3. Alternative CNN Structures

Table 2 lists the performance metrics of the different CNN architectures. The mean value of the macro F1-scores of 5-fold CV is shown here. The performance differences between the three approaches used are minimal. Regardless of the number of CNN blocks and the CNN structure used, the macro F1-score on training (0.935–0.973), validation (0.948–0.96), and test (0.877–0.901) sets are found to be close to the optimum. The performance on the different partial datasets is also very balanced; no significant overfitting can be observed in any of the nine configurations.

### 3.4. Evaluation of the Performance on Different Exercises

The results of the performance evaluation on different datasets are summarized in Table 3. For each dataset, the mean and standard deviation of the macro F1-score from the 5-fold CV are shown.

Several observations can be made using this data: The mean performance on the training, validation and test data is comparable for both DS and TSP, and by far the best in comparison. The comparison between the different sets indicates a slight overfitting on the training data.

The performance of IL combined, IL left and IL right does not vary considerably between each other and no overfitting can be noted. However the performance is significantly worse than the performance of TSP and DS.

Compared to the other exercises, the performance of the HS variants is the poorest. In addition, HS combined, HS left and HS right differ significantly in the performance levels achieved. However, the three variants show no evidence of overfitting.

The calculated standard deviation of the performance metrics is very low for all considered datasets.

### 3.5. Evaluation of the Performance on Unknown Subjects

Table 4 shows the results of the performance test on the data of unknown subjects. A 10-fold LOSOCV was performed for each exercise, and the mean and standard deviation of these 10 runs is shown.

The achieved performance on training and validation dataset is comparable to the one reported in Section 3.4. The exact numbers differ from each other since the weighted F1-score is used in this analysis instead of the macro F1-score. Otherwise, the results are similar: overfitting is only observable to a small extent and the best performance is achieved on the DS and TSP datasets. IL combined, IL right and IL left achieve again similar, but poorer results than TSP and DS. HS combined, HS right and HS left achieve more homogeneous results than before and also reach a significantly higher value.

Striking is the performance on the data of the unknown subject, i.e., on the test set. On average, poor to very poor results are obtained on all exercises, which also scatter significantly.

## 4. Discussion

### 4.1. Measurement Setup

Since we want to develop a universally applicable approach for exercise evaluation in our research, we chose a versatile IMU setup with a large number of sensors. This is the opposite approach of many other research groups [24], who aim to achieve results with a setup as minimal as possible, because smaller setups are preferred in practical use, as they reduce costs and effort. In a future step, the present setup could be reduced for specific application fields. With methods for determining feature importance such as SHAP [46], it could be investigated whether individual IMUs can be excluded from the setup without affecting the performance.

### 4.2. Neural Network Architecture

Our approach to using arbitrarily long repetitions was developed based on the fact that the available labels are assigned to the whole repetition. It is not known which points in time within a repetition are responsible for a certain evaluation. If this information were available, one could follow the HAR approach and divide a repetition into multiple windows, each with its own label. Accordingly, one could work without zero-padding and time-distributed wrappers and also have a larger number of shorter samples. However, the question arises whether a network is still able to evaluate the entire repetition based on an excerpt. The reason for this is the scoring schemes, which differ from exercise to exercise. There are, for example, incorrect movement sequences that only lead to a certain evaluation if they occur in combination. If the network has only one of these segments at its disposal, it does not have the possibility to evaluate such combined criteria.

### 4.3. Hyperparameter Optimization

During the optimization, we have already compared the most common parameters and identified the best possible combination for the application at hand. Yet still it should be considered that this optimization was carried out on the basis of one exercise and a limited subject collective. Accordingly, no statement can yet be made as to whether this parameter combination is also optimal for other exercises. In addition, not every possible parameter combination was checked, but 90 random combinations. The identified configuration thus corresponds to the tendencies that could be read from this subset of possibilities. Decisions for the parameters, as well as for the dataset and the number of combinations tested, were made with feasibility in mind. In order to keep the total computing time within limits, but still obtain as much information as possible, the setup chosen represented a viable middle ground.

### 4.4. Alternative CNN Structures

We compared three different approaches to process the IMU data within the CNN component of the neural network. Overall, no obvious difference in the performance of the three approaches was found. The approaches tested were selected based on the results of Münzner [32] and Rueda et al. [34]. However, it should be checked whether other approaches lead to an improvement of the performance on the present problem. For example, a sensor-specific approach could be tested, which is structured so that separate filters are learned for the accelerometer and gyrometer data. This setup could potentially lead to improved performance and should be investigated.

### 4.5. Evaluation of the Performance on Different Exercises

Depending on the considered FMS exercise, there are significant differences in performance. This behavior will be examined in more detail below.

First, it is noticeable that the results of Section 3.4 and Section 3.5 differ significantly, although the performance on training and validation dataset should be comparable. The described discrepancy can particularly be seen in the case of the HS variants and the IL variants. The reasons for this are the different class weighting of the used evaluation metrics and the class distribution (see Figure 8) of the individual exercise datasets. For example, the HS variants achieved a macro F1-score of 0.582–0.729 on the training dataset but a weighted F1-score of 0.821–0.856. Based on the rating distribution of these variants, one can see that rating “1” is underrepresented, and accordingly, a misclassified example of rating “1” influences the macro F1-score significantly more than misclassified examples of the other classes. In contrast, the influence of a misclassified example on the weighted F1-score is independent of the rating. A good example is the macro F1-score of the HS left dataset: The exercise has a score of 0.582 on the training dataset; at the same time, there are hardly any examples for rating “1”, which therefore has a huge impact on the score.

However, there are also notable differences between the exercises, using the same metric (see Table 3 and Table 4). This phenomenon also occurs in the automatic analysis of FMS exercises with an AdaBoost approach [25]: This approach also delivers the best results on DS and TSP, while IL and HS perform considerably poorer. A possible reason could be the different movement sequences and different evaluation criteria. Some of these criteria are certainly more complex to learn than others, as can be seen in Cook et al. [36]. For example, the evaluation criteria for the DS or TSP could be analyzed by the network using the angles between two IMUs, while criteria for the HS and IL involve multiple body segments at once and would therefore require the combined consideration of four or more IMUs. Especially for these complex criteria, there may be too few examples with too little variation in the datasets to learn a correct representation.

Action should be taken to align HS and IL performance with TSP and DS performance. To compensate for missing examples for certain classes, transfer learning methods could be used. The first processing steps for the analysis of the IMU data should be very similar for all exercises. Accordingly, one could train an appropriately constructed autoencoder with the data of all exercises and use the learned weights of the encoder for the CNN blocks. The higher number of available examples, which also cover a higher variability, could lead to more robust features and thus to increased performance in the evaluation of the different exercises.

### 4.6. Evaluation of the Performance on Unknown Subjects

In this work, we used a LOSOCV split to test the performance of the networks on data from unseen subjects. This led to consistently poor results on the test sets, which also feature considerable variability. Other publications in the field of automatic exercise evaluation encounter similar problems [24,47]. Often, so-called global classifiers are trained on data from all subjects, which then perform reasonably well on a test set of data from those subjects. In addition, “personal” classifiers are trained with the data of one specific subject and perform well on unseen repetitions from that subject [24]. Nevertheless, a considerable number of repetitions is necessary to train these personal classifiers, which must also contain all possible labels. In practice, this will not be possible since a patient will not be able to perform the exercises perfectly at the beginning of a treatment. With the present Deep Learning structure, one should investigate the use of transfer learning techniques to improve the performance. For this purpose, the global classifier is fine-tuned with few repetitions of a new subject. With transfer learning techniques for multivariate timeseries [28], possibly only a few repetitions are necessary, which do not have to contain all possible labels. Appropriate approaches for processing sEMG data [48] and automatic sleep phase [49] detection have already achieved promising results.

### 4.7. Comparison to Classical Machine Learning Methods

Wu et al. [25] have previously presented a system for automated assessment of FMS exercises using IMU data. For the assessment, they use an AdaBoost approach that works with features manually generated from the IMU data. This system achieves a macro F1-score of 0.86 (DS), 0.58 (IL), 0.44 (HS) and 0.86 (TSP) on the test set, without a LOSO split.

Compared to the presented results in Table 3, the neural network achieves better performance than the AdaBoost system. The macro F1-score per exercise is improved by 0.04 (DS), 0.245 (IL), 0.205 (HS), and 0.054 (TSP). However, this comparison should be treated with caution as the used dataset differed in label distribution, measurement setup, and number of subjects and repetitions.

In general, the scalability of the approaches should be considered. Classic machine leaning approaches require hand-crafted features, so transferring them to other exercises always involves additional effort. The deep learning approach presented here represents an end-to-end system that can be applied to other exercises with a suitable dataset (as shown in Section 3.4).

### 4.8. Segmentation

For the intended application, the influence of automated segmentation should also be considered. The exercise repetitions used in this publication are manually separated, but for the practical application, this segmentation must be automated. This automated segmentation could change the characteristics of the data [24]. For example, it would be possible that the automatically segmented repetitions are cut out very precisely, while the previously used repetitions contain some inactivity at the beginning and end. Perhaps the trained network pays special attention to this inactivity and as a result the performance on automated repetitions deteriorates significantly. To avoid this, a segmentation approach such as a k-means algorithm [23] or a Hilbert transform [10] should be used and the influence on the network performance should be analyzed.

## 5. Conclusions

In the presented work, a novel dataset containing approx. 3300 rated repetitions of various FMS exercises was presented.

Based on this dataset, a network structure was developed that allows to classify repetitions into a certain quality category. This network structure is composed of CNN layers, LSTM layers and dense layers. By means of an optimization, different options for the hyperparameters were tested and the best combination was identified.

In addition, the performance of different CNN approaches, designed specifically for the processing of IMU data, was evaluated. The baseline approach proved to be the most useful. Further investigations can be performed in this area, e.g., a sensor-based approach can be tested, which processes the input data ordered by sensor (accelerometer, gyrometer).

The final evaluation of the developed deep learning approach on various exercises shows that the approach is able to classify unknown repetitions from known individuals. However, the performance on unknown data from unknown participants is not sufficient. This problem is already known from other publications. However, the presented deep learning based approach allows the usage of transfer learning methods to address the problem. By means of a few repetitions of a previously unknown subject, it could be possible to adapt the classifier to additional subjects with little training effort.

The evaluation also showed that the performance achieved varies depending on the exercise. Using transfer learning methods, it is likewise possible to develop a classifier that learns more general, robust features, which in turn should lead to more consistent performance.

The presented system lays the foundation for the automated evaluation of physiotherapeutic exercises in the home environment. Such a system increases efficiency and reduces the risk of injury during rehabilitation.

## Figures and Tables

**Table 1:** Table of the parameters that were varied for the hyperparameter optimization, along with the range in which they were varied.

Parameter	Range
Activation function	ReLU/ELU/LReLU
Number of CNN-blocks	1/2/3
Combination scheme for CNN-blocks	increasing filters, fixed kernel size/increasing filters, decreasing kernel size
Regularization technique	dropout (rate 0.2)/batch normalization
Number of LSTM layers	1/2
Batch size	4/8/16/32

**Table 2:** Results of the evaluation of different IMU-specific architectures. Displayed metrics are the arithmetic mean of the macro F1-scores of the 5-fold CV achieved on the training/validation/test dataset.

CNN-Blocks	IMU-Specific (Train/Validation/Test)	Channel-Specific (Train/Validation/Test)	Baseline (Train/Validation/Test)
1	0.945/0.951/0.891	0.96/0.96/0.9	0.936/0.956/0.894
2	0.96/0.958/0.9	0.959/0.948/0.896	0.973/0.959/0.881
3	0.954/0.956/0.896	0.935/0.949/0.877	0.952/0.953/0.901

**Table 3:** Results of the evaluation of the proposed approach on different datasets. For each dataset a 5-fold CV is performed and the arithmetic mean of the macro F1-scores of training, validation and test set is shown, together with the corresponding standard deviation.

Dataset	Training Set	Validation Set	Test Set
Hurdle Step	0.686 ± 0.045	0.679 ± 0.049	0.645 ± 0.049
Hurdle Step right	0.729 ± 0.037	0.755 ± 0.062	0.687 ± 0.041
Hurdle Step left	0.582 ± 0.041	0.566 ± 0.019	0.546 ± 0.018
Inline Lunge	0.877 ± 0.037	0.862 ± 0.05	0.825 ± 0.023
Inline Lunge right	0.863 ± 0.044	0.815 ± 0.062	0.84 ± 0.037
Inline Lunge left	0.868 ± 0.012	0.846 ± 0.05	0.849 ± 0.046
Trunk Stability Pushup	0.953 ± 0.027	0.897 ± 0.043	0.914 ± 0.037
Deep Squat	0.941 ± 0.029	0.948 ± 0.014	0.9 ± 0.021

**Table 4:** Results of the evaluation of the proposed approach on unknown subjects. For each dataset a 10-fold LOSOCV is performed and the arithmetic mean of the weighted F1-scores of training, validation and test set is shown, together with the corresponding standard deviation.

Dataset	Training Set	Validation Set	Test Set
Hurdle Step	0.816 ± 0.019	0.821 ± 0.015	0.301 ± 0.284
Hurdle Step right	0.854 ± 0.04	0.792 ± 0.021	0.267 ± 0.258
Hurdle Step left	0.821 ± 0.019	0.868 ± 0.022	0.405 ± 0.409
Inline Lunge	0.912 ± 0.031	0.88 ± 0.026	0.331 ± 0.177
Inline Lunge right	0.859 ± 0.03	0.806 ± 0.017	0.442 ± 0.353
Inline Lunge left	0.884 ± 0.026	0.813 ± 0.036	0.498 ± 0.347
Trunk Stability Pushup	0.953 ± 0.022	0.954 ± 0.01	0.154 ± 0.318
Deep Squat	0.978 ± 0.01	0.953 ± 0.007	0.485 ± 0.427

## Data Availability

Data is not available due to privacy restrictions.

## Abbreviations

The following abbreviations are used in this manuscript:

FMS	Functional Movement Training
IMU	Inertial Measurement Unit
HAR	Human Activity Recognition
DS	Deep Squat
IL	Inline Lunge
HS	Hurdle Step
TSP	Trunk Stability Pushup
CS	Coordinate System
LOSO	Leave-One-Subject-Out
LOSOCV	Leave-One-Subject-Out Cross Validation
