# Correction: Spilz, A.; Munz, M. Automatic Assessment of Functional Movement Screening Exercises with Deep Learning Architectures. *Sensors* 2023, *23*, 5

**DOI:** 10.3390/s25134110

**Published:** 2025-07-01

**Authors:** Andreas Spilz, Michael Munz

**Affiliations:** Research Group Biomechatronics, University of Applied Sciences Ulm, 89081 Ulm, Germany; andreas.spilz@thu.de

## Text Correction

There was an error in the original publication [1]. We would like to adjust the numerical values in Section 3.2. Specifically, we identified a rounding error in the following sentence, which could lead to a misunderstanding regarding our selection of optimal hyperparameters. This rounding issue might create the impression that we determined the best hyperparameters based on test set performance, which is not the case. Instead, we selected the run with the highest validation performance as the optimal configuration.

A correction has been made to Section 3.2:

The optimal parameter combination is selected using the averaged performance from the 5-fold CV. Considered is the averaged macro F1-score achieved on the validation dataset. The best performance was achieved by a network with the following hyperparameters: batch size 32, three CNN-blocks with the “increasing filter, fixed kernel size” scheme, dropout with a rate of 0.2 as a regularization technique, and two LSTM layers. This combination results in a macro F1-score of 0.956 on the training set, 0.955 on the validation set, and 0.906 on the test set. In all tested configurations, macro F1-scores were achieved in the ranges of 0.932–0.969 (training set), 0.939–0.955 (validation set), and 0.867–0.906 (test set).

The Academic Editor has also instructed us to round all other entries of the same metric in the text to 3 decimal places so that this is consistent throughout the publication. Therefore, there are additional corrections in other sections, which I will list in the following.

A correction has been made to Section 3.3:

Regardless of the number of CNN blocks and the CNN structure used, the macro F1-score on training (0.935–0.973), validation (0.948–0.96), and test (0.877–0.901) sets are found to be close to the optimum.

A correction has been made to Section 4.5:

For example, the HS variants achieved a macro F1-score of 0.582–0.729 on the training dataset but a weighted F1-score of 0.821–0.856. Based on the rating distribution of these variants, one can see that rating “1” is underrepresented, and accordingly, a misclassified example of rating “1” influences the macro F1-score significantly more than misclassified examples of the other classes. In contrast, the influence of a misclassified example on the weighted F1-score is independent of the rating. A good example is the macro F1-score of the HS left dataset: The exercise has a score of 0.582 on the training dataset; at the same time, there are hardly any examples for rating “1”, which therefore has a huge impact on the score.

A correction has been made to Section 4.7:

The macro F1-score per exercise is improved by 0.04 (DS), 0.245 (IL), 0.205 (HS), and 0.054 (TSP).

As the majority of instances of the described metric appear in tables, we also have to add the additional third decimal place to three tables:

A correction has been made to Table 2:
**CNN-Blocks****IMU-Specific** **(Train/Validation/Test)****Channel-Specific** **(Train/Validation/Test)****Baseline** **(Train/Validation/Test)**10.945/0.951/0.8910.96/0.96/0.90.936/0.956/0.89420.96/0.958/0.90.959/0.948/0.8960.973/0.959/0.88130.954/0.956/0.8960.935/0.949/0.8770.952/0.953/0.901

A correction has been made to Table 3:
**Dataset****Training Set****Validation Set****Test Set**Hurdle Step0.686 ± 0.0450.679 ± 0.0490.645 ± 0.049Hurdle Step right0.729 ± 0.0370.755 ± 0.0620.687 ± 0.041Hurdle Step left0.582 ± 0.0410.566 ± 0.0190.546 ± 0.018Inline Lunge0.877 ± 0.0370.862 ± 0.050.825 ± 0.023Inline Lunge right0.863 ± 0.0440.815 ± 0.0620.84 ± 0.037Inline Lunge left0.868 ± 0.0120.846 ± 0.050.849 ± 0.046Trunk Stability Pushup0.953 ± 0.0270.897 ± 0.0430.914 ± 0.037Deep Squat0.941 ± 0.0290.948 ± 0.0140.9 ± 0.021

A correction has been made to Table 4:
**Dataset****Training Set****Validation Set****Test Set**Hurdle Step0.816 ± 0.0190.821 ± 0.0150.301 ± 0.284Hurdle Step right0.854 ± 0.040.792 ± 0.0210.267 ± 0.258Hurdle Step left0.821 ± 0.0190.868 ± 0.0220.405 ± 0.409Inline Lunge0.912 ± 0.0310.88 ± 0.0260.331 ± 0.177Inline Lunge right0.859 ± 0.030.806 ± 0.0170.442 ± 0.353Inline Lunge left0.884 ± 0.0260.813 ± 0.0360.498 ± 0.347Trunk Stability Pushup0.953 ± 0.0220.954 ± 0.010.154 ± 0.318Deep Squat0.978 ± 0.010.953 ± 0.0070.485 ± 0.427

The authors state that the scientific conclusions are unaffected. This correction was approved by the Academic Editor. The original publication has also been updated.
